# The Coordinated Upregulated Expression of Genes Involved in MEP, Chlorophyll, Carotenoid and Tocopherol Pathways, Mirrored the Corresponding Metabolite Contents in Rice Leaves during De-Etiolation

**DOI:** 10.3390/plants10071456

**Published:** 2021-07-16

**Authors:** Xin Jin, Can Baysal, Margit Drapal, Yanmin Sheng, Xin Huang, Wenshu He, Lianxuan Shi, Teresa Capell, Paul D. Fraser, Paul Christou, Changfu Zhu

**Affiliations:** 1 Gansu Provincial Key Laboratory of Aridland Crop Science, Gansu Agricultural University, Lanzhou 730070, China; jinxin9333@163.com; 2 College of Life Science and Technology, Gansu Agricultural University, Lanzhou 730070, China; 3 Department of Plant Production and Forestry Science, University of Lleida-Agrotecnio CERCA Center, Av. Alcalde Rovira Roure, 191, 25198 Lleida, Spain; canbaysal@pvcf.udl.cat (C.B.); xin.huang@udl.cat (X.H.); he.wenshu@udl.cat (W.H.); teresa.capell@udl.cat (T.C.); paul.christou@udl.cat (P.C.); 4 Biochemistry, School of Life Sciences and Environment, Royal Holloway University of London, Egham Hill, Egham, Surrey TW20 0EX, UK; margit.drapal.2011@live.rhul.ac.uk (M.D.); p.fraser@rhul.ac.uk (P.D.F.); 5 School of Life Sciences, Changchun Normal University, Changchun 130032, China; yms_89@sina.com; 6 School of Life Sciences, Northeast Normal University, Changchun 130024, China; lianxuanshi@nenu.edu.cn; 7 ICREA, Catalan Institute for Research and Advanced Studies, Passeig Lluís Companys 23, 08010 Barcelona, Spain

**Keywords:** carotenoids, coordinated gene expression, light-upregulated genes, isoprenoids, rice (*Oryza sativa* L.), secondary metabolites

## Abstract

Light is an essential regulator of many developmental processes in higher plants. We investigated the effect of 4-hydroxy-3-methylbut-2-enyl diphosphate reductase 1/2 genes (*OsHDR1/2*) and isopentenyl diphosphate isomerase 1/2 genes (*OsIPPI1/2*) on the biosynthesis of chlorophylls, carotenoids, and phytosterols in 14-day-old etiolated rice (*Oyza sativa* L.) leaves during de-etiolation. However, little is known about the effect of isoprenoid biosynthesis genes on the corresponding metabolites during the de-etiolation of etiolated rice leaves. The results showed that the levels of α-tocopherol were significantly increased in de-etiolated rice leaves. Similar to 1-deoxy-D-xylulose-5-phosphate synthase 3 gene (*OsDXS3*), both *OsDXS1* and *OsDXS2* genes encode functional 1-deoxy-D-xylulose-5-phosphate synthase (DXS) activities. Their expression patterns and the synthesis of chlorophyll, carotenoid, and tocopherol metabolites suggested that *OsDXS1* is responsible for the biosynthesis of plastidial isoprenoids in de-etiolated rice leaves. The expression analysis of isoprenoid biosynthesis genes revealed that the coordinated expression of the MEP (2-C-methyl-D-erythritol 4-phosphate) pathway, chlorophyll, carotenoid, and tocopherol pathway genes mirrored the changes in the levels of the corresponding metabolites during de-etiolation. The underpinning mechanistic basis of coordinated light-upregulated gene expression was elucidated during the de-etiolation process, specifically the role of light-responsive *cis*-regulatory motifs in the promoter region of these genes. In silico promoter analysis showed that the light-responsive *cis*-regulatory elements presented in all the promoter regions of each light-upregulated gene, providing an important link between observed phenotype during de-etiolation and the molecular machinery controlling expression of these genes.

## 1. Introduction

Plant survival depends on the ability to accurately sense and respond to the extracellular environment at the physiological, biochemical, and molecular levels [1,2]. As an energy source, light is an essential regulator of many developmental processes in higher plants. Besides, light is also involved in regulating the growth and developmental processes of the plant including seed germination, seedling photomorphogenesis, phototropism, gravitropism, chloroplast movement, shade avoidance, circadian rhythms, and flower induction [2,3,4]. Many plants have evolved sophisticated photosensory systems to respond to the extracellular environment appropriately. The effects of light on plant growth and development were particularly evident in the morphology during the de-etiolation process [1,2]. Etiolated *Arabidopsis* (*Arabidopsis thaliana*) seedlings exhibited a characteristic phenotype, such as a pronounced apical hook, elongated hypocotyls, and undifferentiated chloroplasts [5,6]. When etiolated soybean (*Glycine max*) seedlings were exposed to light, the cotyledons expanded, the apical hook opened, and the hypocotyls elongated [7]. A similar phenotype was also observed in etiolated monocotyledonous seedlings. In etiolated rice (*Oryza sativa*), elongated hypocotyls and stem, and rolled and enclosed young leaves were observed [1]. After exposure to light, dramatic morphological changes of etiolated rice seedlings were observed. The leaves expanded and turned green gradually, and apical hooks straightened and opened [1]. During de-etiolation, the morphological changes were accompanied by developmental events, including chloroplast development, pigment synthesis and accumulation, and thylakoid-related photosystem assembly [1]. In *Arabidopsis*, phytochromes, cytochromes, and phytohormones are crucial to regulate the de-etiolation process, individually or cooperatively [2,7]. Chlorophyll biosynthesis is light-dependent, and etiolated seedlings contain non-photosynthetic etioplasts lacking chlorophylls. Carotenoids and the side chains of chlorophylls and tocochromanols are all derived from the MEP (2-C-methyl-D-erythritol 4-phosphate) pathway [8]. When etiolated *Arabidopsis* seedlings are exposed to light, protochlorophyllide IX is rapidly converted to chlorophyllide and then to chlorophylls, resulting in the further formation of thylakoid membranes in the developing chloroplasts [9]. Functional photosystems also require various pigments, chlorophyll *a*/*b*, and carotenoids in photosynthetic reaction centers. Chlorophyll *a*/*b* and carotenoids are crucial to stabilize and assemble newly synthesized photosynthetic complexes [9]. A study by Ghassemian et al. (2006) [9] demonstrated that the amounts of chlorophyll *a*/*b*, β-carotene, lutein, and α-tocopherol significantly increased in *Arabidopsis* seedlings during the de-etiolation process in response to red light. In continuous red light-treated seedlings, mRNA expression and metabolite (e.g., Calvin cycle, biosynthesis of chlorophylls, carotenoids, isoprenoid quinones, thylakoid lipids, sterols, and amino acids) patterns were tightly correlated [9].

The effect of 4-hydroxy-3-methylbut-2-enyl diphosphate (HMBPP) reductase 1/2 genes (*OsHDR1/2*) and isopentenyl diphosphate isomerase 1/2 gene (*OsIPPI1/2*) on the biosynthesis of chlorophylls, carotenoids, and phytosterols in 14-day-old etiolated rice leaves during de-etiolation was investigated, which was characterized by a burst of chlorophyll and carotenoid biosynthesis and accumulation, with no obvious changes in phytosterol accumulation [10]. However, little is known about the effect of other isoprenoid biosynthesis genes involved in mevalonate (MVA) and MEP pathways, chlorophyll, carotenoid, and tocopherol biosynthetic pathways on the corresponding metabolites during the de-etiolation of etiolated monocot plant leaves. Therefore, the comparative expression analysis of the selected isoprenoid pathway genes was carried out between 14-day-old etiolated and de-etiolated rice leaves under different illumination. We further investigated the basis of coordinated light-responsive gene expression, specifically the role of light-responsive *cis*-regulatory motifs in the promoter region of such genes during the de-etiolation process via in silico promoter analysis of all the upregulated genes during de-etiolation.

## 2. Results

### 2.1. Tocochromanol Levels in De-Etiolated Rice Leaves

**Figure 1:**
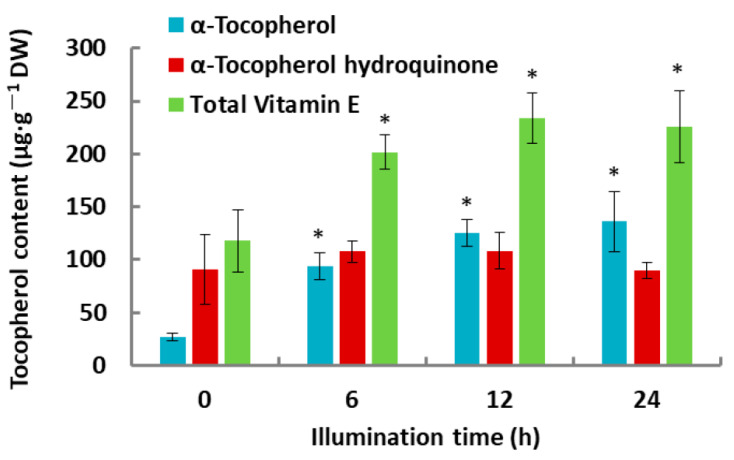
Tocopherol composition and content in the etiolated leaves of 14-day-old rice plants during de-etiolation. Values represent the mean of five biological replicates and bars represent standard deviations. Asterisks indicate statistically significant differences compared with the control (0 h illumination) according to the Student’s *t*-test: * *p* < 0.05.

Tocochromanols were identified and profiled by gas chromatography-mass spectrometry (GC-MS) analysis. Only α-tocopherol and α-tocopherol hydroquinone were detectable. The quantity of α-tocopherol significantly increased in response to illumination (Figure 1). In the first 6 h of illumination, α-tocopherol increased 3.4-fold (from 27.30 ± 3.53 μg·g^−1^ dry weight (DW) to 94. 06 ± 12.42 μg·g^−1^ DW) and then increased up to 5.0-fold compared with that of the dark control after 24 h of illumination. While α-tocopherol hydroquinone amounts exhibited an increasing trend, and no significant differences were detected during de-etiolation.

### 2.2. Functional Analysis of OsDXS Gene Family

**Figure 2:**
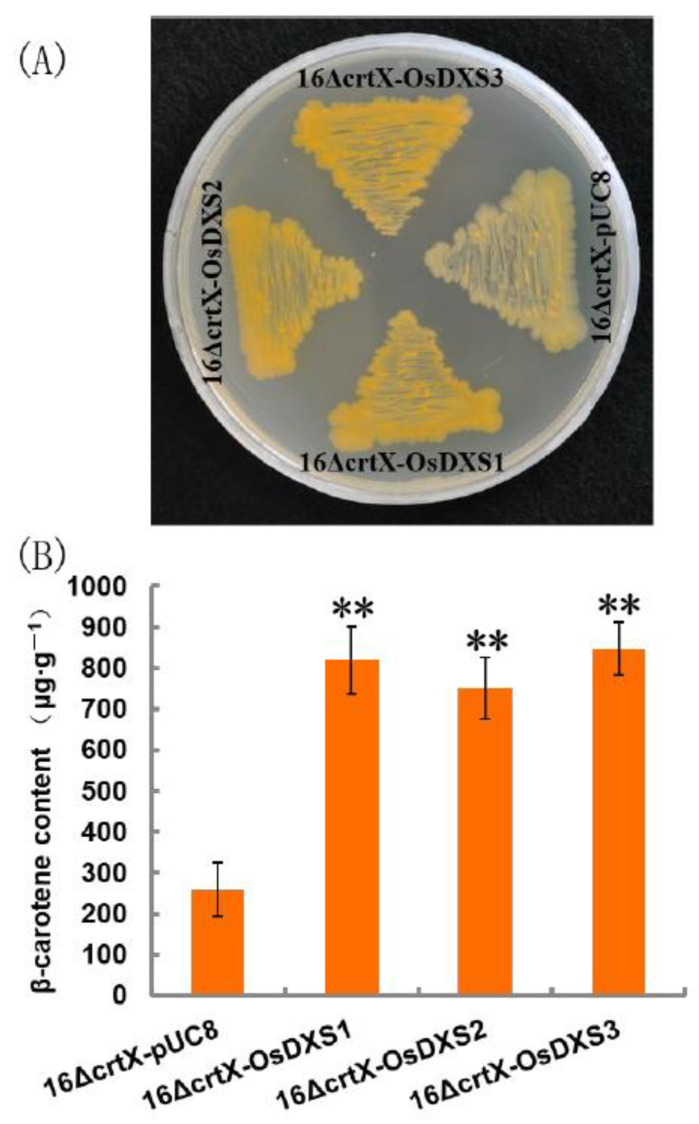
Functional analysis of the *OsDXS* gene family. (**A**) Color complementation and enhanced β-carotene accumulation due to the expression of *OsDXS1*, *OsDXS2*, and *OsDXS3*, respectively, in *E. coli* engineered for β-carotene biosynthesis. The plate was divided into four sections, which were inoculated separately with bacteria carrying pACCAR16∆crtX and pUC8-OsDXS1 (16∆crtX-OsDXS1), pACCAR16∆crtX and pUC8-OsDXS2 (16∆crtX-OsDXS2), pACCAR16∆crtX and pUC8-OsDXS3 (16∆crtX-OsDXS3), or pACCAR16∆crtX and pUC8 (empty vector as a control) (16∆crtX-pUC8). (**B**) Production of β-carotene production by *E. coli* strains expressing *OsDXS1/2/3*. Lane 16∆crtX-pUC8, *E. coli* strain expressing pACCAR16ΔcrtX and pUC8; Lane 16∆crtX-OsDXS1, *E. coli* strain expressing pACCAR16ΔcrtX and pUC8-OsDXS1; Lane 16∆crtX-OsDXS2, *E. coli* strain expressing pACCAR16ΔcrtX and pUC8-OsDXS2; Lane 16∆crtX-OsDXS3, *E. coli* strain expressing pACCAR16ΔcrtX and pUC8-OsDXS3. Values represent the mean of five biological replicates and error bars represent standard deviations. Double asterisks indicate highly significant differences compared with the control (*p* < 0.01, Student’s *t*-test).

In the MEP pathway, pyruvate and glyceraldehyde 3-phosphate are condensed through the action of DXS. DXS is encoded in the rice nuclear genome by three *OsDXS* gene members [11]. *OsDXS3* encoded protein has been demonstrated to have an enzymatic activity that complements the growth of *Escherichia coli* mutant disrupted in the corresponding *DXS* gene [12]. A functional *E. coli* color complementation assay was carried out in a β-carotene accumulating *E. coli* strain to verify enzymatic activity of *OsDXS1* and *OsDXS2* encoded proteins. β-Carotene can be synthesized from foreign carotenogenic genes of pACCAR16ΔcrtX which contains all the carotenogenic genes for the generation of β-carotene from farnesyl pyrophosphate (FPP) in non-carotenogenic *E. coli* as a heterologous host [13]. *E. coli* cells expressing pACCAR16∆crtX and pUC8 were able to accumulate β-carotene when induced by isopropyl β-D-1-thiogalactopyranoside (IPTG) and showed a light-yellow color (Figure 2A). The color of *E. coli* strains co-transformed with pACCAR16∆crtX and pUC8-OsDXS1/2/3 were deeper, reflecting the additional flux provided by OsDXS1/2/3 (Figure 2A). Pigments were extracted from liquid cultures and the β-carotene levels were quantified, revealing that co-transformation with pACCAR16∆crtX and the pUC8 empty vector resulted in a β-carotene yield of 258.99 ± 66.03 μg g^−1^ DW. The addition of pUC8-OsDXS1 resulted in a 3.2-fold increase to 820.47 ± 81.67 μg g^−1^ DW, whereas the addition of pUC8-OsDXS2 and pUC8-OsDXS3 resulted in a 2.9-fold increase to 750.37 ± 75.36 μg g^−1^ DW and 3.3-fold increase to 847.37 ± 63.98 μg g^−1^ DW (Figure 2B). The results indicated that OsDXS1 and OsDXS2 were similar to OsDXS3, encoding functional DXS activities.

### 2.3. Expression Analysis of Genes Involved in MEP and MVA Pathways

**Figure 3:**
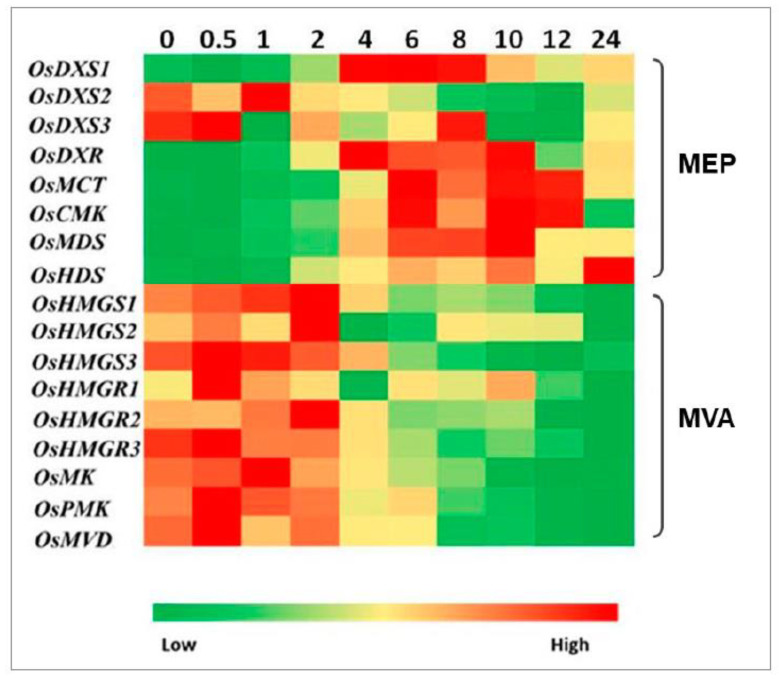
Relative expression levels of the MEP pathway and the MVA pathway genes in etiolated rice leaves during de-etiolation at various times after the onset of irradiation with white light.

The function of *OsDXS1/2/3*, *OsDXR* (DXP reductoisomerase gene), 4-(cytidine 5′-diphospho)-2-C-methyl-D-erythritol kinase gene (*OsCMK*), 2-C-methyl-D-erythritol 2,4-cyclodiphosphate synthase gene (*OsMDS*), and *OsIPPI1/2* encoded proteins has been experimentally confirmed (Figure 2) [4,10,12,14]. The expression profiles of MEP and MVA pathway genes were compared to determine whether the light-triggered the isoprenoid biosynthesis gene expression in rice leaves during the de-etiolation process. The changes in expression levels during illumination were investigated by qRT-PCR separately. Figure 3 illustrates the heat map of comparisons of these gene expressions in rice leaves. The genes in MEP and MVA pathways had different responses to light. The majority of the MEP pathway genes were decreased slightly in the first 0.5 h of illumination, then started to increase until peaks, and after that decreased again. There were exceptions such as *OsDXS2* and *OsDXS3*, and the HMBPP synthase gene (*OsHDS*) among MEP pathway genes. The expression level of *OsDXS1* reached the peak after 6 h illumination, which was four-fold greater compared with that in the dark control. Unlike *OsDXS1*, *OsDXS2* and *OsDXS3* were downregulated after exposure to light, the highest expression level of *OsDXS2* was detected after 1 h (the mRNA level of *OsDXS2* was doubled compared with that at 0.5 h), then kept decreasing until 12 h of illumination and slightly increased from 12 to 24 h of illumination. The transcript abundance of *OsDXS3* was lower than the other two *OsDXS* genes in rice leaves and the mRNA level of *OsDXS3* after 1 h of illumination dropped to one-tenth of the expression level in dark control. From 1 to 8 h, the expression level of *OsDXS3* increased and then decreased sharply (Figure 3).

The expression level of *OsDXR* (DXP reductoisomerase gene) rose rapidly after 2 h irradiation and reached a peak after 4 h of illumination, which was 7.6-fold higher compared with the dark control. From 4 to 10 h of illumination, the expression level of *OsDXR* stayed at a high level and then decreased sharply after 12 h irradiation. The highest expression level of *OsMCT* (2-C-methyl-D-erythritol 4-phosphate cytidylyltransferase gene) was detected after 6 h of irradiation, which was 12-fold higher than that in dark control. After that, the *OsMCT* expression level decreased slightly and stayed at a high level from 8 to 12 h. After 24 h of irradiation, the expression level of *OsMCT* declined rapidly, which was 5.8-fold higher than that in the dark control and a half compared with the peak. The expression pattern of *OsCMK* response to continuous WL (white light) was similar to that of *OsMCT*. The maximum expression level of *OsCMK* was detected at 6 h of illumination (10-fold compared with that in dark control). The expression level of *OsMDS* kept increasing after exposure to WL, and reached the peak after 10 h of illumination, which was 9.9-fold compared to that of the dark control, then decreased to around 6.0-fold after 12 h irradiation. Unlike the rest of the MEP pathway genes, the highest expression level of *OsHDS* (HMBPP synthase gene) was detected after 24 h of illumination (9.8-fold compared to the dark control). The dynamic changes in mRNA levels of the *OsHDR* (HMBPP reductase gene) family have been described by Jin et al. (2020) [10].

The mRNA levels of genes in the MVA pathway during the de-etiolation process were opposite to those in the MEP pathway. The expression levels of MVA pathway genes had higher mRNA levels under dark conditions than those after exposure to light. MVA pathway genes had a similar response after exposure to light. The expression levels of the genes at 10 h of illumination were all lower than those in the dark control. The responses of *OsHMGS* (3-hydroxy-3-methylglutaryl-CoA (HMG-CoA) synthase gene) family genes to WL illumination were not exactly the same. The expression level of *OsHMGS1* was increased slightly in the first 2 h of illumination, then decreased sharply from 2 to 6 h, and the expression level of *OsHMGS1* was stable at a low level until 10 h (less than half compared with that in dark control). Similar to *OsHMGS1*, the mRNA level of *OsHMGS2* was stable and kept at a low level after 4 h of illumination. The expression level of *OsHMGS3* started to decrease after 1 h of illumination, and the lowest expression level was detected after 12 h, which was only 0.08-fold compared with that in the dark control. The *OsHMGR* (HMG-CoA reductase gene) family also showed distinct reactions to continuous WL illumination. The maximum expression levels of *OsHMGR1-3* were detected at 0.5, 2, and 0.5 h of illumination, respectively. The expression level of *OsHMGR1* dropped by 50% compared with the dark control after 24 h of illumination. The mRNA level of *OsHMGR2* and *OsHMGR3* in the dark control was 7.9-fold and 18-fold greater than that after 24 h of illumination, respectively (Figure 3). In the first 1 h, the expression level of *OsMK* (mevalonate kinase gene) increased slightly, then decreased until 24 h. The expression level of *OsMK* in the dark control was 3.4-fold higher than that after 24 h illumination. The mRNA level of *OsPMK* (phospho-MVA kinase gene) and *OsMVD* (MVA diphosphate decarboxylase gene) both increased slightly in the first 0.5 h of irradiation, and after that kept decreasing. The expression changes in the *OsIPPI* family, involved in both MVA and MEP pathways, were described by Jin et al. (2020) [10].

### 2.4. Expression Analysis of Genes Involved in Carotenoid, Tocopherol, and Chlorophyll Biosynthetic Pathways

**Figure 4:**
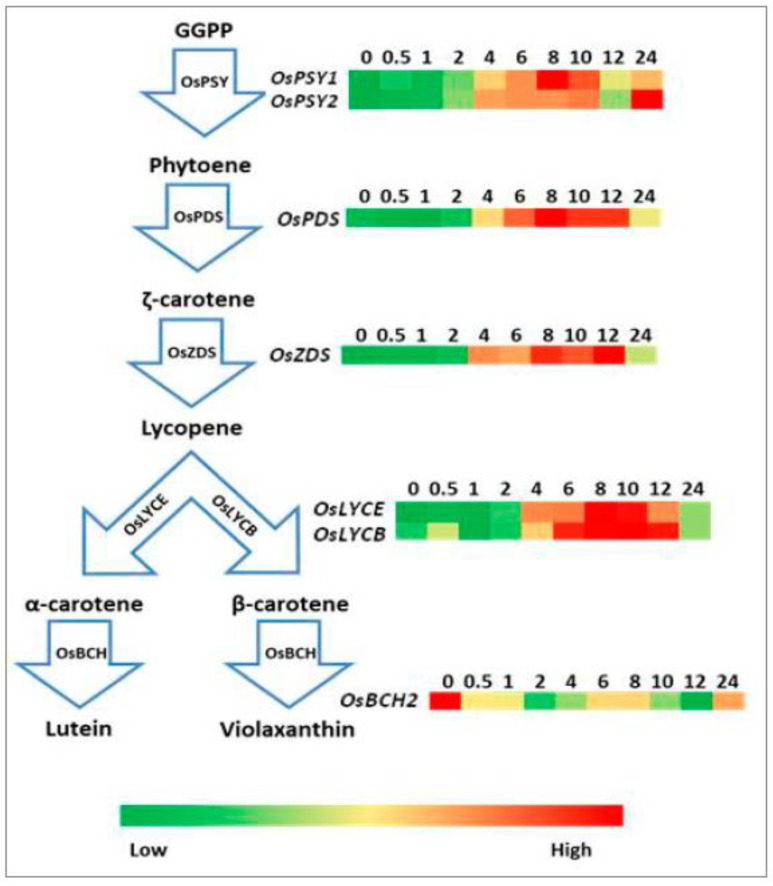
Relative expression levels of carotenoid pathway genes in etiolated rice leaves during de-etiolation at different times after the onset of irradiation with white light.

The function of rice phytoene synthase 2 (*OsPSY2*), phytoene desaturase (*OsPDS*), ζ-carotene desaturase (*OsZDS*), and lycopene β-cyclase (*OsLYCB*) has been demonstrated [15,16]. The expression levels of the selected genes related to the carotenoid biosynthetic pathway are shown in Figure 4. The expression level of both *OsPSY1* and *OsPSY2* increased immediately after exposure to WL illumination. The expression level of both *OsPSY1* and *OsPSY2* reached high levels after 8 and 10 h of illumination (approximately 5- and 25-fold, respectively, greater than the expression level in the dark control), respectively, but both decreased sharply after 10 h of illumination. The expression of *OsPSY1* remained nearly constant from 12 to 24 h of illumination, while the expression of *OsPSY2* increased rapidly again after 12 h of illumination and reached its maximum after 24 h. The expression level of *OsPDS* decreased slightly during the first 1 h of illumination, then increased to the peak after 8 h of illumination (4.1-fold compared with that in the dark control). After the peak, the expression level of *OsPDS* declined until 24 h, when its expression level was 2.4-fold higher than that of the dark control. The expression level of *OsZDS* kept increasing in the first 12 h of illumination and then declined at 24 h (8.5- and 4.5-fold higher than the dark control). The mRNA levels of both *OsLYCB* and *OsLYCE* (lycopene ε-cyclase gene) reached their peaks after 8 h of illumination (4.7- and 2.3-fold compared to the dark control, respectively). Unlike the other genes, *OsBCH2* (β-carotene hydroxylase 2 gene) showed a decrease in expression level after exposure to WL, which decreased to about 50% of that of the dark control after 24 h of illumination (Figure 4).

**Figure 5:**
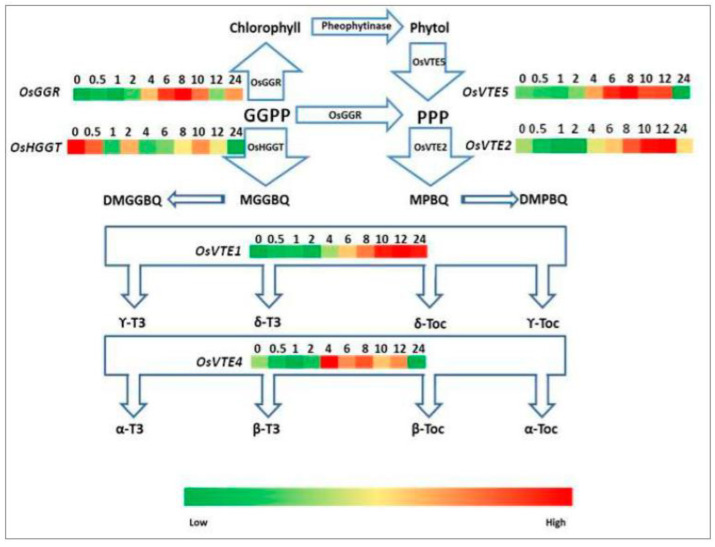
Relative expression levels of tocopherol pathway genes in etiolated leaves during de-etiolation at different times after the onset of irradiation with white light. Abbreviations: DMGGBQ, 2,3-dimethyl-6-geranylgeranylbenzoquinol; DMPBQ, 2,3-dimethyl-6-phytylbenzoquinol; MGGBQ, 2-methyl-6-geranylgeranylbenzoquinol; MPBQ, 2-methyl-6-phytylbenzoquinol; *OsGGR*, geranylgeranyl reductase gene; *OsHGGT*, rice homogentisate geranylgeranyltransferase gene; *OsVTE1*, tocopherol cyclase gene; *OsVTE2*, homogentisate phytyltransferase gene; *OsVTE4*, γ-tocopherol methyltransferase gene; *OsVTE5*, phytol kinase gene; PPP, phytyl pyrophosphate; α-Toc, α-Tocopherol; β-Toc, β-Tocopherol; γ-Toc, γ-Tocopherol; δ-Toc, δ-Tocopherol; α-T3, α-Tocotrienol; β-T3, β-Tocotrienol; γ-T3, γ-Tocotrienol; δ-T3, δ-Tocotrienol.

The key tocochromanol biosynthesis genes in rice have been identified [17], while the function of these genes remained undetermined. The expression levels of the selected tocopherol biosynthetic pathway genes in de-etiolated rice leaves are shown in Figure 5. The expression levels of *OsGGR* (geranylgeranyl reductase gene), *OsVTE5* (phytol kinase gene), *OsVTE2* (homogentisate phytyltransferase gene), *OsVTE1* (tocopherol cyclase gene), and *OsVTE4* (γ-tocopherol methyltransferase gene) were all upregulated by WL. The expression levels of *OsGGR* increased rapidly during the first 8 h of illumination (10-fold greater than that of dark control) and then plummeted after 12 h of irradiation. The expression level of *OsGGR* mRNA remained on the rise from 12 to 24 h of illumination. The mRNA of *OsVTE1* remained at low levels during the first 2 h of illumination, then started to increase to its maximum after 12 h of illumination (4.9-fold higher than that of dark control), and remained at this high level until 24 h irradiation. There were no significant differences in expression levels among 10-, 12-, and 24-h illuminated samples. Both *OsVTE5* and *OsVTE2* mRNA levels exhibited a downward trend at the beginning, then started to increase and reached a peak respectively at 8 and 12 h (2.5- and 2.0-fold higher than that in the dark control, respectively). Afterward, the expression levels started to decline, *OsVTE5* mRNA level decreased sharply after 24 h of illumination, and the *OsVTE2* mRNA level dropped to a level similar to that in the dark control after 24 h irradiation. The expression level of *OsVTE4* increased rapidly during the first 4 h of illumination and decreased until 24 h of illumination. However, *OsHGGT* (homogentisate geranylgeranyltransferase gene) was downregulated by WL. The expression level decreased to three-fold greater than the dark control after 24 h of illumination.

**Figure 6:**
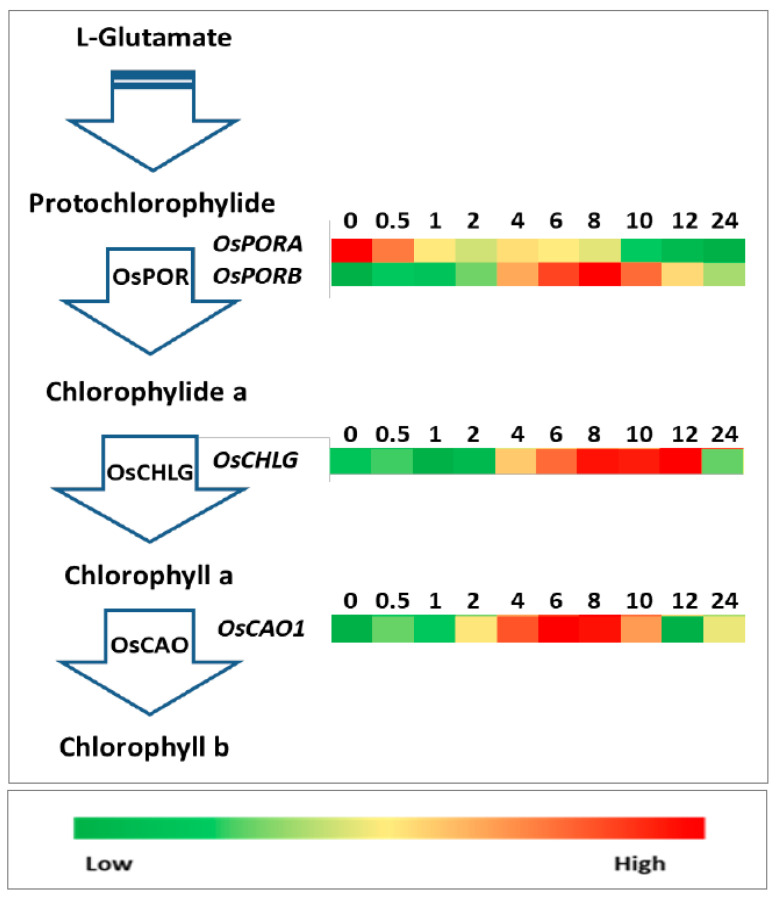
Relative expression levels of chlorophyll biosynthesis pathway genes in etiolated rice leaves during de-etiolation at different times after the onset of irradiation with white light.

So far, the function of a few chlorophyll biosynthesis genes (rice chlorophyll synthase gene (*OsCHLG*) and rice chlorophyll *a* oxygenase 1 gene (*OsCAO1*)) has been demonstrated [4,18,19]. Changes in the expression levels of the selected chlorophyll biosynthetic pathway genes during rice de-etiolation are shown in Figure 6. The expression levels of *OsPORA* (protochlorophyllide oxidoreductase A gene) and *OsPORB* reacted differently to WL illumination. The *OsPORB* mRNA level increased significantly in the first 8 h (15-fold higher than that in dark control) and decreased rapidly after 24 h of illumination. The expression level at 24 h was approximately three-fold greater than in the dark control. However, the expression of *OsPORA* started to decline after exposure to WL, indicating that *OsPORA* was downregulated by light. The expression of *OsCHLG* increased in the first 0.5 h of illumination, decreased slightly after 2 h of illumination, increased again after 4 h of irradiation, and remained stable from 8 to 12 h. The maximum expression level was 4.5-fold higher than that in the dark control, which was detected after 12 h irradiation. After that, *OsCHLG* expression decreased immediately to the same level as that in the dark control. The expression level of *OsCAO1* reached a peak after 6 h of illumination (8.4-fold), decreased until 12 h, and slightly increased again from 12 to 24 h.

### 2.5. In Silico Promoter Analysis of Light-Responsive Cis-Regulatory Elements in the Promoter Regions of Light-Upregulated Genes

The full-length CDS (coding sequence) of each rice (*O*. *sativa* cultivar: Nipponbare) gene was used to identify its corresponding promoter region from NCBI. The mRNA with the longest 5′-untranslated region and its corresponding promoter sequence (up to 1.0 kb upstream of transcription start site) of each upregulated gene during de-etiolation of etiolated rice leaves were retrieved from NCBI (Table 1). The online tools PlantCare [20] and PLACE [21] were used for the identification of light-responsive *cis*-acting regulatory elements in the promoter of each gene. The promoters of the upregulated genes (*OsDXS1*, *OsDXR*, *OsMCT*, *OsCMK*, *OsMDS*, *OsHDS*, *OsHDR1*, *OsIPPI1*, *OsPORB*, *OsCHLG*, *OsCAO1*, *OsPSY1*, *OsPSY2*, *OsPDS*, *OsZDS*, *OsLYCB*, *OsLYCE*, *OsGGR*, *OsVTE1*, *OsVTE2*, *OsVTE4*, *OsVTE5*) during de-etiolation of etiolated rice leaves contained light-upregulated elements, including G-box (CACGTC), IBOXCORE (I-box; GATAA), Box 4 (ATTAAT), GT1-motif (GGTTAA), MRE (AACCTAA), G-box (TAAACGTG), Sp1 (GGGCGG), LTR (CCGAAA), G-box (CCACGTAA), TCCC-motif (TCTCCCT), G-box (GTCGTG), Box II (CCACGTGGC), AE-box (AGAAACAA), G-box (CGCGTC), G-box (GCCACGTGGA), G-box (CACGTT), GATA-motif (GATAGGG), GATA-motif (GATAGGA), and TCT-motif (TCTTAC) (Table 1).

## 3. Discussion

### 3.1. The Coordinated Expression Levels of the MEP and MVA Pathway Genes and Carotenoid, Tocopherol and Chlorophyll Biosynthetic Pathway Genes Mirrored the Levels of the Corresponding Metabolite Changes during De-Etiolation

The analysis of *Arabidopsis* revealed that the genes involved in the MVA and MEP pathways behaved differently in response to light stimulation. The expression levels of MVA pathway genes were inhibited by light, whereas those of MEP pathway genes were upregulated [8]. In rice, Kim et al. (2005) [11] reported that, in the case of light illumination, only the relative expression of *OsDXS1* increased relative to the unilluminated control. OsDXS3 has been proposed to be specifically involved in the synthesis of diterpenoid phytoalexins (such as momilactones and phytocassanes) in suspension-cultured rice cells after treatment with a chitin elicitor [12,22].

Carotenoids, tocopherols, and chlorophylls are synthesized primarily from precursors derived from the MEP pathway [8]. The number of carotenoids and chlorophylls increased significantly in response to the illumination of etiolated rice seedlings compared with that of the dark control [10]. The quantity of α-tocopherol significantly increased (Figure 1). The first step in the MEP pathway is catalyzed by DXS. There are three *OsDXS* genes in the rice genome. The expression of *OsDXS1* increased four-fold after 4 h of illumination, whereas the expression levels of *OsDXS2* and *OsDXS3* decreased after exposure to light (Figure 3). Similar to *OsDXS3* [12], both the encoded OsDXS1 and OsDXS2 are shown to be functional enzymes (Figure 2) in engineered *E. coli*. The appearance time of MEP-derived isoprenoids in the de-etiolated rice leaves coincided with the time of the positive response of *OsDXS1* to light (Figure 3; Supplementary Figure S1), suggesting that the biosynthesis of chlorophylls, carotenoids, and tocopherols in the de-etiolated rice leaves is mainly generated by OsDXS1. The rest of the MEP pathway gene (*OsDXR*, *OsMCT*, *OsCMK*, *OsMDS*, *OsHDS*, and *OsHDR1*) expression (Figure 3; Jin et al., 2020 [10] for *OsHDR1*) had positive correlations with light illumination correlated with the MEP pathway-derived carotenoid, chlorophyll, and tocopherol accumulation. A study by Huang et al. (2018) reported photosynthetic pigments in an *OsMDS* mutant (*IspF*), which displayed a yellow-green leaf phenotype, and the chlorophyll and carotenoid levels were significantly lower than those in WT rice. Therefore, *OsMDS* is essential for the biosynthesis of chlorophylls and carotenoids [14]. The genes encoding enzymes involved in chlorophyll biosynthesis, such as OsCHLG, OsPORB, and OsCAO1, were upregulated by light, except OsPORA (Figure 6), which was associated with the accumulation of chlorophylls.

Phytoene synthase (PSY) is the rate-limiting enzyme of carotenogenesis. In *Arabidopsis* seedlings, the expression level of *PSY* increased under the continuous illumination of both FR and red light [23]. In white mustard (*Sinapis alba* L.), the upregulation of *PSY* expression was observed after light exposure, while *PDS* and *GGPS* (geranylgeranyl pyrophosphate synthase gene) expression levels remained constant [24]. Both rice *OsPSY1* and *OsPSY2* are subject to phytochrome and involved in carotenoid biosynthesis in photosynthetically active tissues with different expression patterns during chloroplast development [25]. Carotenoid biosynthesis genes exhibit high expression in light-grown rice tissues [26]. In agreement with reports by Welsch et al. (2008) and Chaudhary et al. (2010) [25,26], the expression levels of *OsPSY1/2*, *OsPDS*, *OsZDS*, *OsLYCB*, and *OsLYCE* increased after exposure to WL in this study, whereas the *OsBCH2* expression level decreased relative to that of the dark control (Figure 4), reflecting the approximately two-fold increases in the abundance of β-carotene after 24 h of illumination compared to that of the dark control [10].

Studies on *Arabidopsis* found that AtVTE2 showed limited activities in tocopherol synthesis [27]. *OsVTE2* was upregulated by light. Both *OsVTE1* and *OsVTE5* were also upregulated by WL in de-etiolated rice leaves (Figure 5). Meanwhile, *OsVTE4* increased rapidly in the early stage of rice de-etiolation. The upregulation of tocopherol biosynthetic genes in de-etiolated rice leaves was reflected by the increase in tocopherol content in rice leaves during de-etiolation.

Phytosterols are derived from the MVA pathway. In a previous study [10], the level of β-sitosterol increased significantly in the first 6 h of light illumination and then decreased after 12 h of illumination to the same level as that of the dark control. After 24 h of light illumination, the levels of campesterol and stigmasterol showed no difference compared with that of the dark control [10]. The expression levels of the selected MVA pathway genes were inhibited with no obvious changes during the de-etiolated rice leaves (Figure 3), consistent with the MVA pathway-derived phytosterol changes.

### 3.2. The Underpinning Mechanistic Basis of Coordinated Light-Responsive Gene Expression during the De-Etiolation Process

The coordinated expression of light-responsive genes during the de-etiolation can reflect similarities between their promoters. A range of light-responsive elements (LREs) has been documented in different promoters, many of which positively or negatively mediate gene expression in response to light [3]. Although many LREs and their transcription factors have been identified, no single element is found in all light-upregulated gene promoters, suggesting a complex light-regulation network and a lack of a universal switch. It has been suggested that combinations of LREs, rather than individual elements, could confer proper light-responsiveness to a light-insensitive basal promoter [3,28]. To investigate the basis of coordinated light-responsive gene expression during the de-etiolation of rice etiolated leaves, in silico promoter analysis of upregulated genes during de-etiolation was performed. Most promoters contained at least two motifs involved in the response to light, but both *OsCMK* and *OsHDR1* promoters (Table 1) had only one light-responsive motif (IBOXCORE, GATAA), supporting that these genes were upregulated by light. However, surprisingly, except for *OsCMT*, *OsLYCE*, and *OsVTE2* promoters, other light-upregulated gene promoters all presented a *cis*-regulatory element with a consensus sequence GATAA (IBOXCORE) (Table 1). IBOXCORE (I-box) is a conserved sequence upstream of light-upregulated genes for both monocots and dicots [21,28]. (http://www.dna.affrc.go.jp/htdocs/PLACE/; 18 March 2020 (See IBOX, S000199)).

## 4. Materials and Methods

### 4.1. Plant Material

Wild type rice (*Oryza sativa* L. cv. EYI105) seeds were sterilized in 3.7% NaClO for 20 min and washed with sterilized water thoroughly, then were aseptically grown on Murashige and Skoog (MS) medium containing 0.3% phytagel in pots (diameter 10 cm, height 20 cm, 9 seeds in each pot) in a controlled growth chamber at 25 °C in complete darkness. Fourteen-day-old etiolated rice seedlings were illuminated with continuous WL (100 μmol m^−2^ s^−1^) for 0.5, 1, 2, 4, 6, 8, 10, 12, or 24 h (hour). Leaves of de-etiolated rice seedlings were rapidly frozen in liquid nitrogen after collection and stored at −80 °C. The mixture of all the rice leaves from the same pot was collected and stored as one sample.

### 4.2. Tocochromanols Analysis

Rice leaf samples were freeze-dried before analysis, and 10 mg of powdered tissue was extracted using a chloroform-methanol mixture, as described [10]. The gas chromatography-mass spectrometry (GC-MS) method was applied to determine tocochromanols as described in detail previously [29]. Compositions of tocochromanols were identified using a mass spectral library built from in-house standards [30]. The samples used for tocochromanols analysis were from the same batch [10] as those used for carotenoids, chlorophylls, phytosterols, and pathway gene expression analysis.

### 4.3. Rice DXS cDNA Cloning and Functional Complementation Analysis

The *OsDXS1*, *OsDXS2*, and *OsDXS3* cDNAs were cloned directly from rice leaf mRNA by RT-PCR using two pairs of oligonucleotide primers with terminal EcoRI and BamHI restriction sites for both *OsDXS1* and *OsDXS3*, or EcoRI and HindIII restriction sites for *OsDXS2* (Supplementary Table S2). The primers were designed to flank the open reading frame (ORF) of each gene according to sequences in GenBank (accession number XM_015785019 for *OsDXS1*, XM_015787004 for *OsDXS2*, and XM_015792458 for *OsDXS3*). The RT-PCR products were transferred to vector pCRII TOPO (Invitrogen, Carlsbad, CA, USA) to generate pCR-OsDXS1, pCR-OsDXS2, and pCROsDXS3 for sequencing. The *OsDXS1*, *OsDXS2*, and *OsDXS3* cDNAs were then introduced into pUC8 at the EcoRI/BamHI or EcoRI/HindIII sites to generate vectors pUC8-OsDXS1, pUC8-OsDXS2, and pUC8-OsDXS3 for functional complementation in engineered *E. coli* cells. The integrity of all intermediate and final constructs was confirmed by sequencing.

Functional color complementation experiments were conducted as previously described [10,13]. *E. coli* DH5α cells were transformed with pACCAR16ΔcrtX, which contains the *Erwinia uredovora* carotene biosynthesis genes *crtE*, *crtB*, *crtI*, and *crtY* encoding geranylgeranyl pyrophosphate (GGPP) synthase, phytoene synthase, phytoene desaturase, and lycopene β-cyclase, respectively, for the generation of β-carotene [31]. β-Carotene levels in engineered *E. coli* were measured as described previously [10].

### 4.4. Gene Expression Analysis with Quantitative Real-Time PCR (qRT-PCR)

Total RNA was isolated using the RNeasy Plant Mini Kit (Qiagen, Hilden, Germany) and DNA was removed with DNase I (RNase-Free DNase Set, Qiagen) [10]. First-strand cDNA was synthesized from 1 µg total RNA using Ominiscript Reverse Transcriptase (Qiagen) in a 20 µL total reaction, and quantitative real-time RT-PCR (qRT-PCR) was applied [10,32] using the primers listed in Supplementary Table S1. The amplified DNA fragments for each gene were confirmed by sequencing. The expression levels of the genes in different samples were normalized against rice actin mRNA (*OsActin*). Three biological replicates each comprising three technical replicates were tested for each sample.

### 4.5. Retrieval of Promoter Regions and Analysis of Cis-Regulatory Elements

Gene and promoter sequences (1.0 kb upstream of transcription start site) of each rice (*O*. *sativa* cultivar: Nipponbare) gene under consideration were retrieved from NCBI (*National Center for Biotechnology Information*) (https://www.ncbi.nlm.nih.gov/; accessed on 18 March 2020). The tools PlantCare (http://bioinformatics.psb.ugent.be/webtools/plantcare/html/; accessed on 18 March 2020) [20] and PLACE (http://www.dna.affrc.go.jp/htdocs/PLACE/; 18 March 2020) [21] were used to identify *cis*-regulatory elements in promoters.

### 4.6. Data and Statistical Analysis

Significant differences in quantitative dependent variables for any two alternative treatments were calculated using Student’s *t*-test, with a *p*-value of 0.05 considered significant.

## 5. Conclusions

Both *OsDXS1* and *OsDXS2* genes were shown to encode functional DXS enzymes. The appearance of MEP-derived plastidial isoprenoids in the de-etiolated rice leaves correlates well with the observed upregulation of *OsDXS1* expression, suggesting that OsDXS1 is responsible for the biosynthesis of chlorophylls, carotenoids, and tocopherols in the de-etiolated rice leaves. The coordinated upregulated expression of genes involved in the MEP pathway, chlorophyll, carotenoid, and tocopherol biosynthetic pathways mirrored the increased levels of chlorophyll, carotenoid, and tocopherol contents in rice leaves during de-etiolation, demonstrating that the synthesis of these plastidial metabolites is under transcriptional control. Moreover, the work was extended to elucidate the underpinning mechanistic basis of coordinated light-responsive gene expression during the de-etiolation process. The light-responsive *cis*-regulatory elements were presented in all promoter regions (up to 1.0 kb upstream of transcription start site) of each light-upregulated gene, serving as a base for the further characterization of these light-induced genes during the de-etiolation process.

## Figures and Tables

**Table 1:** In-silico analysis of light-responsive *cis*-acting regulatory elements of upregulated genes during de-etiolation of etiolated rice leaves. The positions of the *cis*-acting regulatory elements are relative to those of cDNA (+1 is the first nucleotide of cDNA).

Gene Name	*Cis*-Acting Regulatory Elements Involved in Light Responsiveness	GenBank Accession Numbers
*OsDXS1*	G-box (CACGTC, -102); IBOXCORE (I box; GATAA, -422, -485, -510); Box 4 (ATTAAT, -501, -748); GT1-motif (GGTTAA, -990)	XM_015785019 (mRNA); AP014961 (gDNA, genomic DNA)
*OsDXR*	G-box (CACGTT, -52); IBOXCORE (GATAA, -200, -233); Box 4 (ATTAAT, -664); MRE (AACCTAA, -852)	AK099702 (mRNA); AP014957 (gDNA)
*OsMCT*	G-box (TAAACGTG, -35); Box 4 (ATTAAT, -54); Sp1 (GGGCGG, -819, -952)	XM_015771410 (mRNA); AP003346 (gDNA)
*OsCMK*	IBOXCORE (GATAA, -552)	XM_015766647 (mRNA); AP003221 (gDNA)
*OsMDS*	IBOXCORE (GATAA, -384, -564, -667); GT1-motif (GGTTAA, -857)	NC_029257 (mRNA); AP005823 (gDNA)
*OsHDS*	G-box (CACGAC, -46); IBOXCORE (GATAA, -980)	XM_015770238 (mRNA); AP004124 (gDNA)
*OsHDR1*	IBOXCORE (GATAA, -17)	XM_015777111 (mRNA); AC103550 (gDNA)
*OsIPPI1*	G-box (CACGTG, -143); LTR (CCGAAA, -278); IBOXCORE (GATAA, -898); G-box (CACGAC, -974)	XM_015791312 (mRNA); AP014963 (gDNA)
*OsPORB*	Box II (CCACGTGGC, -55); AE-box (AGAAACAA, -143); IBOXCORE (GATAA, -156); G-box (CGCGTC, -514); G-box (GCCACGTGGA, -557); G-box (CACGTT, -566); GATA-motif (GATAGGG, -628)	XM_015759459 (mRNA); AC068923 (gDNA)
*OsCHLG*	GATA-motif (GATAGGA, -189); TCT-motif (TCTTAC, -297); IBOXCORE (GATAA, -978)	XM_015782164 (mRNA); AC136221 (gDNA)
*OsCAO1*	LTR (CCGAAA, -841, -810); Box 4 (ATTAAT, -749, -697); G-box (CACGTC, -519, -8); Box II (CCACGTGGC, -489); TCT-motif (TCTTAC, -358); IBOXCORE (I-box; GATAA, -70)	XM_015758600 (mRNA); AP014966 (gDNA)
*OsPSY1*	TCCC-motif (TCTCCCT, -24); G-box (CACGTC, -30); IBOXCORE (GATAA, -70, -926); Box 4 (ATTAAT, -464, -590, -749); AE-box (AGAAACAA, -877)	XM_015787027 (mRNA); AP005750 (gDNA)
*OsPSY2*	G-box (CACGTG, -86, -92); Box 4 (ATTAAT, -183, -464, -630); IBOXCORE (GATAA, -940)	AK073290 (mRNA); AL831803 (gDNA)
*OsPDS*	G-box (TACGTG, -173); IBOXCORE (GATAA, -341, -379); G-box (CACGAC, -864)	XM_015777615 (mRNA); AC079633 (gDNA)
*OsZDS*	IBOXCORE (GATAA, -66, -538, -569); TCCC-motif (TCTCCCT, -314); Box 4 (ATTAAT, -902)	XM_015791038 (mRNA); AP004273 (gDNA)
*OsLYCB*	Box 4 (ATTAAT, -171, -569); IBOXCORE (GATAA, -634); GT1-motif (GGTTAA, -738); G-box (CACGTT, -769)	XM_015771748 (mRNA); AP005849 (gDNA)
*OsLYCE*	Sp1 (GGGCGG, -115, -815); GT1-motif (GGTTAA, -288); G-box (CACGTC, -376)	XM_015766712 (mRNA); AP003332 (gDNA)
*OsGGR*	MRE (AACCTAA, -22); IBOXCORE (GATAA, -348, -457); GT1-motif (GGTTAA, -612); GT1-motif (GGTTAAT, -836)	XM_015771626 (mRNA); AP004114 (gDNA)
*OsVTE1*	G-box (CCACGTAA, -74); Sp1 (GGGCGG, -156); G-box (CACGTG, -184); IBOXCORE (GATAA, -498, -573)	XM_015770843 (mRNA); AP005536 (gDNA)
*OsVTE2*	Box 4 (ATTAAT, -275); MRE (AACCTAA, -916)	XM_015789025 (mRNA); AP005760 (gDNA)
*OsVTE4*	Box 4 (ATTAAT, -87); IBOXCORE (GATAA, -746, -809)	XM_015767600 (mRNA); AP003994 (gDNA)
*OsVTE5*	AE-box (AGAAACAA, -817); IBOXCORE (GATAA, -846)	XM_015769509 (mRNA); AP003252 (gDNA)

Abbreviations: *OsDXS1*, rice 1-deoxy-D-xylulose-5-phosphate (DXP) synthase 1 gene; *OsDXR*, rice DXP reductoisomerase gene; *OsMCT*, rice 2-*C*-methyl-D-erythritol 4-phosphate cytidylyltransferase gene; *OsCMK*, rice 4-(cytidine 5′-diphospho)-2-*C*-methyl-D-erythritol kinase gene; *OsMDS*, rice 2-*C*-methyl-D-erythritol 2,4-cyclodiphosphate synthase gene; *OsHDS*, rice 4-hydroxy-3-methylbut-2-enyldiphosphate (HMBPP) synthase gene; *OsHDR1*, rice HMBPP reductase 1 gene; *OsIPPI1*, rice isopentenyl diphosphate isomerase 1 gene; *OsPORB*, rice protochlorophyllide oxidoreductase B gene; *OsCHLG*, rice chlorophyll synthase gene; *OsCAO1*, rice chlorophyll *a* oxygenase 1 gene; *OsPSY1/2*, rice phytoene synthase 1/2 genes; *OsPDS*, rice phytoene desaturase gene; *OsZDS*, rice zeta-carotene desaturase gene; *OsLYCE*, rice lycopene epsilon cyclase gene; *OsLYCB*, rice lycopene beta-cyclase gene; *OsGGR*, rice geranylgeranyl reductase gene; *OsVTE1*, rice tocopherol cyclase gene; *OsVTE2*, rice homogentisate phytyltransferase gene; *OsVTE4*, rice gamma-tocopherol methyltransferase gene; *OsVTE5*, rice phytol kinase gene.

## Data Availability

Not applicable.

## Supplementary Materials

The following are available online at https://www.mdpi.com/article/10.3390/plants10071456/s1, Additional Supporting Information may be found in the online version of this article: Figure S1. Expression of *OsDXS1*, *OsDXS2*, and *OsDXS3* in etiolated rice (*Oryza sativa*, EYI105) leaves during de-etiolation at different times after the onset of irradiation with white light, with mRNA levels normalized against *OsActin* mRNA. Table S1. Primers used for qRT-PCR analysis in this study. Table S2. Primers used for rice *DXS* cDNA cloning in this study.
