# Supplementary material for: The Coordinated Upregulated Expression of Genes Involved in MEP, Chlorophyll, Carotenoid and Tocopherol Pathways, Mirrored the Corresponding Metabolite Contents in Rice Leaves during De-Etiolation
Source: Plants (Basel). 2021 Jul 16;10(7):1456. doi: 10.3390/plants10071456 (PMC8309317; doi:10.3390/plants10071456)
Supplement: Supplementary file 1 [file plants-10-01456-s001.zip › plants-1279072-supplementary.pdf]

plants

MDPI

Article

The Coordinated Upregulated Expression of Genes Involved in MEP, Chlorophyll, Carotenoid and Tocopherol Pathways, Mirrored the Corresponding Metabolite Contents in Rice Leaves during De-Etiolation

Xin Jin [1,2,3,†], Can Baysal [3,†], Margit Drapal [4], Yanmin Sheng [5], Xin Huang [3], Wenshu He [3], Lianxuan Shi [6], Teresa Capell [3], Paul D. Fraser [4], Paul Christou [3,7] and Changfu Zhu [3,5,*]

1 Gansu Provincial Key Laboratory of Aridland Crop Science, Gansu Agricultural University, Lanzhou 730070, China
2 College of Life Science and Technology, Gansu Agricultural University, Lanzhou 730070, China
3 Department of Plant Production and Forestry Science, University of Lleida-Agrotecnio CERCA Center, Av. Alcalde Rovira Roure, 191, 25198 Lleida, Spain
4 Biochemistry, Royal Holloway University of London, Egham Hill, Egham, Surrey TW20 0EX, UK
5 School of Life Sciences, Changchun Normal University, Changchun 130032, China
6 School of Life Sciences, Northeast Normal University, Changchun 130024, China
7 ICREA, Catalan Institute for Research and Advanced Studies, Passeig Lluís Companys 23, 08010 Barcelona, Spain
* Correspondence: changfu.zhu@udl.cat
† These authors contributed equally to this work.

Citation: Jin, X.; Baysal, C.; Drapal, M.; Sheng, Y.; Huang, X.; He, W.; Shi, L.; Capell, T.; Fraser, P.D.; Christou, P.; et al. The Coordinated Upregulated Expression of Genes Involved in MEP, Chlorophyll, Carotenoid and Tocopherol Pathways, Mirrored the Corresponding Metabolite Contents in Rice Leaves during De-Etiolation. *Plants* **2021**, *10*, 1456. https://doi.org/10.3390/plants10071456

Academic Editor: Fabrizio Araniti

Received: 14 June 2021
Accepted: 8 July 2021
Published: 16 July 2021

Publisher's Note: MDPI stays neu-tral with regard to jurisdictional claims in published maps and institu-tional affiliations.

Copyright: © 2021 by the authors. Licensee MDPI, Basel, Switzerland. This article is an open access article distributed under the terms and conditions of the Creative Commons Attribution (CC BY) license (http://creativecommons.org/licenses/by/4.0/).

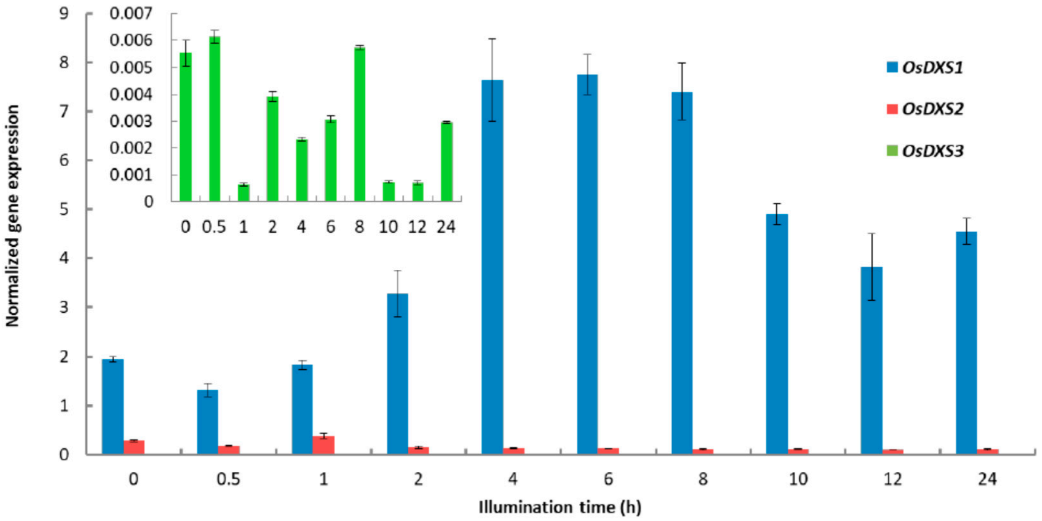

Figure S1. Expression of *OsDXS1*, *OsDXS2* and *OsDXS3* in etiolated rice (*Oryza sativa*, EYI105) leaves during de-etiolation at different times after the onset of irradiation with white light, with mRNA levels normalized against *OsActin* mRNA. Three biological replicates and three technical replicates were tested. Bars indicate±SD.

Table S1. List of primers used for the qRT-PCR.

Gene	Forward primer (5′- 3′)	Reverse primer (5′- 3′)
OsActin	GACTCTGGTGATGGTGTCAGC	TCATGTCCCTCACAATTT
OsDXS1	CTCAAGGGAGGGAAGAACAA	ACACCTGCTTGTTGTCGTTG
OsDXS2	TGTTGTGGAGCTCGCTATTG	TCCTCCCACCTAGATCCCTT
OsDXS3	ACCTCCTCGGGAAGAAGAAG	GAGGGACACCTGCTTGTTGT
OsDXR	TCCACCCACAATCTATCA	GGTGACCTCTGAGCAATA
OsMCT	GACTTGAGGTCACTGATG	AGCAAGTCATCAGGAGTC
OsCMK	GTATTTGCAGCATGCGTCGT	ACCTGGTGCTTTCTACTCACAG
OsMDS	TCGTCGGTGTTCATGAGG	TTGTCTCCTTGAATGGGC
OsHDS	GGGTTGCATTGTCAATGG	CTGAATCAAGGCGTCAGT
OsCHLG	CTGACTTCTTTGTATAGCATAGCTGG	CTGCAACGGATAATTGAGTTATGTCA
OsPORA	CGGCCTCGTCTGAGTTTATTAT	CCTCTCTCACTGAAAGCTGAAA
OsPORB	GTGAGTGAGAGTGATGTGCTATT	CTCCTCCATCGATCTTTCTTGG
OsCAO1	GGGAGACCTGGATGTGTTATG	ATGGTAAGGGCATTGGATTCT
OsPSY1	GAGGCGGGAAGAGGAGATAAA	GGAAGGCAGATGCTGTCTGATT
OsPSY2	TTCTTTTGATTTCGCACGATTTC	TGGCAGGCTTATGGCATACA
OsPDS	GATCCAAACCGTTCAATGCTGG	TGTCACTCCGTCCAACCCATT
OsZDS	GCTGCCCTTTTTGCCTTGA	CACAATAACGAAGAACAC ACCATTG
OsLYCB	CGTCCAGTACGACAAGCCGTA	AAGGGCATGGCGTAGAGGAACG
OsLYCE	GTATGGCAGGGTTCACAGGGAC	GCCAGCGTCATAGCATCGTCTC
OsBCH2	TCGAGAACGTGCCCTACTTCC	ACCCACCTCCTCCAACTCCTT
OsHGGT	ATGGGGATTTCTTGAGGCTTTATC	GGCTGGGCTTATTGACCTTATC

OsGGR	GCACCCGAGGCCTAAGAGGG	GAAGTAGATCCCCTCGCCGGAG
OsVTE1	ATAACTTTTGGGGAAGTAGGCATG	AAACTCGACTAGAAAATTCCTGAG
OsVTE2	TCACTGGTTTGCTGGAGGCAG	AAGAGTTGGCTTGTTAACCTTATC
OsVTE4	GTCACTGTCTCTTGAGGATATAAG	TCTTATCGTCTTCCACCCACTTC
OsVTE5	GCGGGGTCTTCGAGCAGAAAC	GCATAGCTTCCTGACCTGAAAAG
OsHMGS1	TTGTTGCCTCCTGGGACGTT	GATCTCCTCGTCGGCCTTCC
OsHMGS2	GCCTACGCCTTCCTCCCAAT	ATGCCCACGTCCTTCCTCTC
OsHMGS3	GGGATGGACGCTACGGTCTT	TAGCAGCAGCACCACCTGTT
OsHMGR1	TGGGCTTGTACATACTGCTGA	AACCTGACCTGCTGCTATCC
OsHMGR2	ACCCAAGAGCTTCACATGCT	TCCGGAGTTAGCGACTTTGT
OsHMGR3	TCCTGTGAAATGGGTGGCTTT	ACCGGTTTCATGACTCAGCA
OsMK	CACTAGTGGATCGCGACCGT	TGGCCTGCACAATGCAAACT
OsPMK	ACTTGGGTCATCAGCTGCCA	GATCTCGTCCAGCTGCGTTG
OsMVD	CCGTCAACGACAGCATCAGC	ATCTCCTTGCCGTTGAGCCA

Abbreviations: ***OsDXS1*/2/3**, rice (*Oryza sativa*) 1-deoxy-D-xylulose-5-phosphate synthase 1/2/3 genes; ***OsDXR***, rice 1-deoxy-D-xylulose 5-phosphate reductoisomerase gene; ***OsMCT***, rice MEP cytidyltransferase gene; ***OsCMK***, rice CDP-ME kinase gene; ***OsMDS***, rice 2-C-methyl-D-erythritol 2,4-cyclodiphosphate (ME-2,4cPP) synthase gene; ***OsHDS***, rice 1-hydroxy-2-methyl-2-butenyl 4-diphosphate (HMBPP) synthase gene; ***OsCHLG***, rice chlorophyll synthase gene; ***OsPORA*,** rice protochlorophyllide oxidoreductase A gene; ***OsPORB***, rice protochlorophyllide oxidoreductase B gene; ***OsCAO1*,** chlorophyllide *a* oxygenase 1 gene; ***OsPSY1*/2**, rice phytoene synthase 1/2 genes; ***OsPDS***, rice phytoene desaturase gene; ***OsZDS***, rice ζ-carotene desaturase gene; ***OsLYCB***, rice lycopene β-cyclase gene; ***OsLYCE***, rice lycopene ε-cyclase gene; ***OsBCH2***, rice β-carotene hydroxylase gene; ***OsHGGT***, rice homogentisate geranylgeranyltransferase gene; ***OsVTE1***, rice tocopherol cyclase gene; ***OsVTE2***, rice homogentisate phytyltransferase gene; ***OsVTE4***, rice γ-tocopherol methyltransferase gene; ***OsVTE5***, rice phytol kinase gene; ***OsHMGS1*/2/3**, rice HMG synthase 1/2/3 genes; ***OsHMGR1*/2/3**, rice 3-hydroxy-3-methylglutaryl-CoA reductase 1/2/3 genes; ***OsMK***, rice MVA kinase gene; ***OsPMK***, rice phospho-MVA kinase gene; ***OsMVD***, rice MVA diphosphate decarboxylase gene.

Table S2. Primers used for rice *DXS* cDNA cloning in this study.

Gene	Forward primer (5′- 3′)	Reverse primer (5′- 3′)
OsDXS1	GAATTCCATGGCGCTCACGACGTTCTCC ATTTCGAGAGGAGG	GGATCCTACGCGTTGGGCACCGTCATGA TGGCGAGCGCCT
OsDXS2	GAATTCCATGGGTGGGAGATATCTGCAT TCTCCACCTGCTGTTA	AAGCTTCCTTCTTACTTCATCAACAAAA GTGCGTCTCGGTGTC
OsDXS3	GAATTCCATGGGTGGGAGATATCTGCAT TCTCCACCTGCTGTTA	GGATCCTCAGCTGAGCTGAAGTGCCTCC AATGGCCTCCCAAG

EcoRI (GAATTC), BamHI (GGATCC) and HindIII (AAGCTT) restriction sites are underlined. Abbreviations: ***OsDXS1*/2/3**, rice (*Oryza sativa*) 1-deoxy-D-xylulose-5-phosphate synthase 1/2/3 genes
